# Inflammatory Proteomic Network Architecture of Visceral Adiposity and Cardiometabolic Traits

**DOI:** 10.21203/rs.3.rs-11156776/v1

**Published:** 2026-09-28

**Authors:** Raul Mendez-Giraldez, Cong Wang, Ruisheng Wang, Jacob Joseph, Meryl S. LeBoff, I-Min Lee, Howard D. Sesso, Xuehong Zhang, JoAnn E. Manson, Edward Giovannucci, Deirdre K. Tobias, Azam Yazdani

**Affiliations:** 1. Independent Researcher, Boston, MA; 2. Division of Preventive Medicine, Department of Medicine, Brigham and Women’s Hospital, Boston, MA; 3. Channing Division of Network Medicine, Department of Medicine, Brigham and Women’s Hospital, Boston, MA; 4. Harvard Medical School; 5. U.S. Department of Veterans Affairs and the VA Boston Healthcare System; 6. Division Endocrinology, Diabetes, and Hypertension, Department of Medicine, Brigham and Women’s Hospital, Boston, MA; 7. School of Nursing, Yale University, New Haven, Connecticut; 8. Department of Epidemiology, Harvard TH Chan School of Public Health, Boston, MA

## Abstract

**Background:**

Visceral adipose tissue (VAT) is strongly associated with cardiometabolic disease, but the network architecture connecting visceral adiposity to systemic inflammatory proteins and cardiometabolic traits remains poorly characterized.

**Methods:**

We applied an artificial intelligence (AI)-driven network integration approach to plasma inflammatory proteomics (Olink Explore 384) and cardiometabolic traits (VAT, HOMA-IR, glucose, systolic blood pressure [SBP], triglycerides [TG], LDL-C, and HDL-C) in the Vitamin D and Omega-3 Trial (VITAL) discovery cohort (n = 635). Network findings were evaluated for replication in the independent Cocoa Supplement and Multivitamin Outcomes Study (COSMOS) cohort (n = 371). Within-person associations between 2-year changes in proteins and corresponding clinical traits were also evaluated in VITAL.

**Results:**

VAT emerged as the central network hub, exhibiting direct connections with multiple cardiometabolic traits and more direct protein connections than any other trait. No inflammatory proteins were directly connected to glucose, HOMA-IR, or SBP in the integrated network; instead, their network connectivity with the inflammatory proteome was dependent on VAT. A triangular network motif linking VAT, HGF, and IL1RN was identified and independently replicated in COSMOS. Longitudinal analyses further showed that 2-year changes in VAT tracked with changes in HGF and IL1RN, while changes in HGF tracked with changes in IL1RN. Overall, 7 of 10 direct protein–trait connections identified at baseline remained significant longitudinally. PON3 exhibited connectivity with both VAT and HDL-C, with opposite-direction associations that remained significant longitudinally (VAT: *β* = −0.97, *p* = 8 × 10^−4^; HDL-C: *β* = 1.44, *p* = 4× 10^−7^).

**Conclusion:**

Visceral adiposity occupies a central position within the integrated inflammatory proteomic and cardiometabolic network. The reproducibility of the VAT–HGF–IL1RN network motif across cohorts and over time, together with the longitudinal stability of most protein–trait connections, supports a VAT-centered organization of inflammatory and cardiometabolic relationships. These findings provide a network-based framework for investigating molecular connections between visceral adiposity, lipid metabolism, and systemic inflammation.

## Introduction

Unraveling the molecular architecture that links visceral adiposity to systemic metabolic decline requires moving beyond isolated biomarker associations. While visceral adipose tissue (VAT) is well-established as a pathogenic, immuno-metabolic hub that drives chronic inflammation through the release of cytokines and free fatty acids into the portal system,^1-3^ the precise structural dependencies within these pathways remain obscure. Traditional epidemiological frameworks, including univariable and linear regression strategies, typically evaluate inflammatory proteins and clinical traits individually. Although these single-biomarker approaches are highly effective for initial risk stratification, they are inherently limited; they cannot capture the multi-protein covariance structures, topological hierarchies, or complex networks that define biological systems.

In our recent analysis using conventional epidemiological and network-based approaches, we identified distinct inflammatory proteomic signatures associated with VAT and other cardiometabolic traits and found that accounting for VAT substantially attenuated many of these associations.^4^ These findings provided evidence that visceral adiposity may play an important role in the relationship between circulating inflammation and cardiometabolic traits, but they did not resolve the underlying multivariable network structure or determine how proteins and traits are conditionally connected within this system.

Consequently, a critical question remains unanswered: whether systemic inflammatory patterns track with diverse cardiometabolic traits along independent, parallel axes or whether their joint variance is structurally anchored around visceral adiposity. While traditional clinical metrics capture the systemic consequences of metabolic dysfunction, high-throughput proteomics provides an opportunity to resolve the underlying molecular architecture of these relationships.

The present study builds on the artificial intelligence (AI)-driven network integration approach^5^ to reconstruct the interdependencies between the circulating proteome and cardiometabolic traits. This approach addresses heterogeneity, high dimensionality, and incomplete data across participants by first learning relationships within each data layer and subsequently integrating the two layers to learn connections between the proteome and clinical traits, enabling us to test whether VAT occupies a central position within the integrated network.

Using high-throughput plasma proteomics and dual-energy X-ray absorptiometry (DXA)-quantified VAT together with a broad spectrum of cardiometabolic traits, we sought to define the overarching network topology linking visceral adiposity, inflammatory proteins, and metabolic dysfunction.

We validated the stability and generalizability of this network architecture using a rigorous discovery-and-replication design. The initial network discovery was conducted within the Vitamin D and Omega-3 Trial cohort (VITAL,^6^ n=635), followed by targeted replication of key topological dependencies and direct protein–trait links in the independent Cocoa Supplement and Multivitamin Outcomes Study (COSMOS5,^7^ n=371). Crucially, because cross-sectional snapshots cannot capture dynamic biomarker tracking, we complemented our network discovery by analyzing longitudinal, within-person proteomic fluctuations over time. This dual-cohort, longitudinal framework provides a rigorous test of whether temporal shifts in the inflammatory proteome mirror concordant changes in the clinical cardiometabolic trait, establishing the identified network structure as a robust, reproducible biological feature.

## Results

The baseline characteristics of the discovery and replication cohorts are summarized in Table 1. Both cohorts have similar BMI and approximately 50% female participants. However, participants in the replication cohort are, on average, 5 years older and have a 10% higher proportion of White participants compared to the discovery cohort. The cardiometabolic traits evaluated included VAT, systolic blood pressure (SBP), fasting glucose, triglycerides (TG), HDL-C, and HOMA-IR.

Following quality control (QC), 355 circulating inflammatory proteins were evaluated. An independent proteomic network was first reconstructed using the full discovery sample (n = 635). Concurrently, an independent clinical trait network was reconstructed within the subset of participants with baseline VAT quantification (n = 525). The proteomic and clinical trait layers were then integrated using the overlapping participants (n = 525) to evaluate edge connectivity (Methods). The resulting integrated network distinguishes conditional dependencies that remain after accounting for other variables.

Visualization of the proteomic network after ForceAtlas2^8^ spatial organization revealed three major densely interconnected subnetworks. Based on the predominant biological functions of the proteins in each cluster, these visually distinct clusters were annotated as representing intracellular regulation/signaling, immune–metabolic inflammation, and extracellular matrix (ECM) organization and tissue remodeling. The intracellular regulation/signaling cluster exhibited limited direct connections with clinical traits. Conversely, all cardiometabolic traits, including VAT, were directly associated with proteins in the immune–metabolic inflammatory cluster or were linked indirectly through VAT-dependent network pathways.

Circulating proteins demonstrated a higher frequency of direct edge connections with VAT than with any other clinical traits within the integrated network architecture. Consistent with this topology, the clinical traits HOMA-IR, SBP, and fasting glucose lacked direct edge connections to the proteomic layer. Instead, the network paths linking these clinical endpoints to the inflammatory proteome were dependent on VAT.

Low-density lipoprotein cholesterol (LDL-C) exhibited a distinct topological position within the network, exhibiting edge connectivity along two distinct pathways: one via connections with HDL-C through two proteins, and another through independent edge connections with intracellular regulatory proteins.

The p-values and effect sizes for proteins directly linked to the traits are provided in Table 2. HGF (hepatocyte growth factor) and AGRP (agouti-related peptide) were identified as protein hubs with high connectivity, exhibiting direct associations with VAT and TG, respectively. Among the proteins directly associated with VAT, HGF and IL1RN were also directly connected within the proteomic network, forming a triangular network structure with VAT (Figure 1B). IL32 (interleukin-32) showed a direct association with HDL-C and had five direct protein–protein connections (connectivity = 5), whereas SIT1 exhibited a direct association with HDL-C but no direct protein–protein connections (connectivity = 0) at the significance threshold of α = 1 × 10^−3^. The expanded network neighborhoods of these trait-linked proteins are shown in Figure 2.

Within VAT–HGF–IL1RN network structure, HGF, and IL1RN exhibited strong direct associations with VAT, with effect sizes of 0.55 (SE = 0.03, p = 3 × 10^−42^) for HGF and 0.59 (SE = 0.03, p = 4 × 10^−49^) for IL1RN. In terms of broader network architecture, HGF had six direct protein–protein connections, while IL1RN had three. This specific VAT–HGF–IL1RN structure was subsequently evaluated for replication in the independent COSMOS cohort and for longitudinal consistency using within-person changes across all three network components (see the sections below).

### Replication

The connectivity among the clinical traits (trait-trait network edges) observed in VITAL was replicated in the independent COSMOS cohort (n = 371; α = 1 × 10^−3^), with the sole exception of the direct relationship between SBP and VAT (Figure 3), which replicated at a relaxed threshold of α = 1 × 10^−2^.

We next evaluated whether the direct protein–trait connections identified in the VITAL discovery cohort were preserved in the COSMOS replication cohort. Notably, out of the 10 direct protein–trait connections identified in VITAL, 7 replicated in the COSMOS cohort (Figure 3), including the links between HGF and IL1RN to VAT, AGRP to TG, and IL32 to HDL-C. The direct HGF–IL1RN protein–protein connection was also replicated, resulting in preservation of the VAT–HGF–IL1RN triangular network structure in the independent cohort.

### Variability in protein-trait associations over time

To assess the temporal stability of baseline protein–trait relationships, we examined whether within-person changes in trait-linked proteins tracked corresponding changes in associated traits over time (Table 3, Figure 4). Of the 10 direct protein–trait associations identified at baseline, 7 remained significant longitudinally (p < 0.05).

The strongest longitudinal associations were observed for AGRP with TG (β = 0.86, SE = 0.08, p = 1×10^−23^), PON3 with HDL-C (β = 1.44, SE = 0.28, p = 4×10^−7^), and HGF with VAT (β = 0.67, SE = 0.17, p = 1×10^−4^). Significant longitudinal associations were also observed for PON3 with VAT, as well as for IL1RN, PSPN, and SIT1 with their corresponding baseline traits. In contrast, longitudinal associations involving WFIKKN2, SLC39A5, and IL32 did not reach statistical significance.

Overall, the majority of direct protein–trait associations identified at baseline (7 of 10) were also observed longitudinally, with within-person changes in protein levels paralleling changes in their corresponding traits. Changes in IL1RN were also significantly associated with changes in HGF (β = 0.19, SE = 0.02, *p* = 2.5 × 10^−13^), a part of the triangular motif.

## Methods

### Data availability.

The discovery cohort for deriving inflammatory proteomic markers included general population adults from the US-based Vitamin D and Omega-3 Trial.^6^ Our replication cohort is from the Cocoa Supplement and Multivitamin Outcomes Study (COSMOS, n = 371).^7^ Requests to access the dataset from qualified researchers trained in human subject confidentiality protocols should be sent to the Steering Committees of the parent trials.

### Cohorts.

VITAL was a double-blind, placebo-controlled randomized trial to evaluate the effects of vitamin D and marine omega-3 fatty acid supplementation for the primary prevention of cancer and cardiovascular disease, initiated in November 2011 with active intervention and follow-up concluding in December 2017 (NCT01169259).^6^ COSMOS was a double-blind, placebo-controlled randomized trial evaluating the effects of cocoa extract and multivitamin supplements for the prevention of cancer and cardiovascular disease, with an active intervention period spanning from June 2015 through December 2020 (NCT02422745).^7^

In both studies, a Boston-area subcohort of participants without cardiovascular disease or cancer underwent detailed in-person baseline phenotyping at the Clinical and Translational Science Center (CTSC) in Boston, MA, including DXA whole-body scans and biospecimen collection. Eligible participants were men aged ≥ 50 years and women aged ≥ 55 years who were free of cardiovascular disease and cancer at baseline. Individuals were excluded if they had a history of renal failure or severe hepatic dysfunction. Both studies were conducted in accordance with the Declaration of Helsinki; all participants provided written informed consent at enrollment, and the sub-cohort protocols were approved by the Mass General Brigham Institutional Review Board (Boston, MA).

### DXA.

Visceral adiposity was estimated using full-body DXA scans, a low-radiation alternative to computed tomography (CT) that provides comparable quantification.^9^ Clinical fat depots were measured in a 5 cm abdominal region above the iliac crest, corresponding to the CT measurement area. The software automatically identifies the outer and inner abdominal wall margins at the level of the fourth lumbar vertebra and measures total fat mass within this region, which includes both subcutaneous and visceral fat. Subcutaneous fat above and below the visceral region is then estimated and subtracted from the total abdominal fat to derive VAT mass.

### Cardiometabolic traits.

We examined cardiometabolic traits, including SBP, HDL-C, LDL-C, TG, fasting glucose, and insulin resistance (HOMA-IR). The traits HDL-C, LDL-C, glucose, and TG were determined from fasting blood samples obtained from in-clinic assessments. Oral glucose tolerance testing was performed, and the HOMA-IR index was calculated from fasting insulin and glucose values.^10^ SBP was assessed using direct blood pressure measurement during physical examination.^11^

### Proteomics measurements.

Plasma proteins were quantified using the *Olink Explore 384 platform (Olink Proteomics, Uppsala, Sweden)*, which measures 384 inflammation-related proteins using proximity extension assay (PEA) technology. In this assay, pairs of antibodies conjugated to complementary oligonucleotides bind to their target protein, allowing the oligonucleotides to hybridize and generate a unique DNA template that is subsequently amplified and quantified by next-generation sequencing. To ensure assay performance, the platform incorporates internal controls that monitor incubation, extension, and amplification steps, together with external negative, plate, and sample controls. For each assay plate, the limit of detection (LOD) is calculated from triplicate negative controls. Protein abundance was expressed as normalized protein expression (NPX), a relative quantification metric obtained through extension-control normalization, log2 transformation, and inter-plate normalization.

For each protein, measurements below the LOD were flagged by the manufacturer as "under LOD." Following the manufacturer's recommendations, these observations were retained in all downstream analyses rather than excluded. Lower and upper limits of quantification (LLOQ and ULOQ) were analyte-specific and are provided in the manufacturer's assay documentation. The analytical performance of the Olink Explore 384 platform has been extensively validated, with reported average intra-assay coefficients of variation of approximately 8–11% and inter-assay coefficients of variation of approximately 15–25%, demonstrating high assay reproducibility.

### Proteomics QC.

QC and preprocessing of the inflammatory proteomic data were executed via a multistage quality-control pipeline: 1) Sample-level filtering excluded all samples flagged with platform QC warnings. 2) Protein-level filtering excluded proteins with measurements below the protein-specific LOD in more than 50% of samples. 3) For the remaining proteins, continuous Normalized Protein Expression (NPX) values reported below the LOD were retained. This approach preserves the observed variability in NPX values and is consistent with the manufacturer’s recommendation to retain continuous measurements below the LOD. 4) Continuous protein levels were winsorized to the 5th and 95th percentiles to reduce the influence of extreme values while preserving the overall distribution of the data. 5) Finally, all protein values were standardized to a mean of 0 and a standard deviation of 1 to ensure effect sizes were directly comparable across the panel. Following QC, 355 proteins were retained for downstream analyses.

### Analytical Approach

#### Proteomics and trait network integration.

To formally integrate the circulating proteome with clinical traits, we applied the AI-Driven Network Integration approach.^7^ This method operates on the premise that in cross-sectional studies, each omics layer may already reflect existing variation in the other omics layers. We therefore assume that the network structure within each omics layer can be estimated independently before identifying cross-omics connectivity:

(1) $$str_{layer\;i}⊥str_{layer\;j},\;for\;\forall \;i\neq j,$$

where the structure (str) of omics i is independent of the structure of omics j, for all i not equal to j. Operationally, we executed this integration in a two-stage sequential workflow to handle platform heterogeneity, high dimensionality, and missing data across layers:

1. Within-layer network reconstruction: We first reconstructed network structures within each layer (the proteome and the clinical traits) utilizing the maximum available sample size for each respective dataset.
2. Between-layer network integration: We subsequently leveraged the overlapping samples across datasets to learn and map the edge connectivity between the two layers, thereby resolving the overarching topological hierarchy linking the circulating proteins to the clinical traits.

Both network structures at each layer of data were identified using the PC algorithm, a probabilistic graphical-model approach for identifying conditional dependencies among variables using Gaussian partial correlations,^12^ where the edges between conditionally independent variables are removed. To identify the proteomic network, we normalized and then adjusted the proteins for batch effects (as fixed effects), sex, age, and race. We identified the network structure of proteomics using n = 635 samples. We also normalized traits and then adjusted them for batch effects, sex, age, and race. We uncovered the underlying connectivity among traits using n = 525 samples (due to excluding the participants without VAT measurement). Finally, we extended the PC algorithm under the assumption reviewed in formula (1) and integrated the proteomics network with the trait network to uncover the connectivity between proteomics and traits.

We selected a common sparsity parameter of α = 1 × 10^−3^ for the proteomic, clinical-trait, and integrated networks because it provided a balance between network sparsity and connectivity, allowing sufficient structure to be retained for biological interpretation without producing overly dense networks. We subsequently evaluated a range of sparsity parameters using the SPOT^13^ (Sparse Optimal Topology) method as a data-driven assessment of network topology (Figure 5). SPOT identified α = 1 × 10^−3^ as the inflection point at which the topology of the integrated network changed most substantially, providing independent support for the selected sparsity level.

Visualization of the network using the ForceAtlas2 layout^8^ revealed three visually distinct regions of densely interconnected proteins. Functional annotations were assigned by reviewing Gene Ontology, Reactome, UniProt, and published literature.

#### Replication in the COSMOS cohort.

To replicate the direct connectivity of proteins on specific traits, we took the same approach as the discovery cohort. We first adjusted traits for covariates sex, age, race, and batch effects, and then identified conditional (in)dependence properties of the traits as well as proteins and traits at α = 1 × 10^−3^.

#### Change analysis.

Focusing on protein-trait direct associations in the discovery cohort, we fit a linear regression of delta trait (year 2 trait – baseline trait) on delta protein (year 2 protein – baseline protein) and adjusted models for trait level at baseline, active vitamin D, and fish oil. In a secondary analysis, we also adjusted models for age at baseline and race. However, the results did not differ significantly from those of the first model.

## Discussion

We found that VAT had more direct connections with inflammatory proteins than any other cardiometabolic trait, positioning visceral adiposity as a central hub linking systemic inflammation with cardiometabolic risk. In contrast, no inflammatory proteins were directly connected to SBP, glucose, or HOMA-IR, suggesting that their associations with the inflammatory proteome may be mediated primarily through VAT within the inferred network. Most VAT-associated proteins were replicated in COSMOS and, except WFIKKN2, showed significant longitudinal associations with changes in VAT, supporting the robustness of these network relationships across populations and over time. Notably, the six proteins associated with VAT, including PSPN, WFIKKN2, PON3, HGF, IL1RN, and SLC39A5, occupied distinct network modules representing different biological pathways, such as inflammation and extracellular matrix remodeling. Their convergence on VAT suggests that visceral adiposity may connect cardiometabolic risk to multiple biological pathways rather than a single inflammatory pathway.

Within this network, we identified a VAT–HGF–IL1RN triangular motif, with HGF and IL1RN directly connected to VAT and to each other. This topology was independently replicated in COSMOS, while longitudinal analyses showed that VAT changes tracked with changes in both HGF and IL1RN, and changes in HGF tracked with changes in IL1RN. HGF, a pleiotropic growth factor involved in tissue repair, remodeling, and metabolic regulation, has been linked to visceral adiposity, cardiovascular disease, and malignancy^14^. Together, these findings nominate the VAT–HGF–IL1RN axis as a potential molecular link between visceral adiposity and systemic inflammatory biology.

PON3 emerged as another distinctive network node, exhibiting strong longitudinal stability through a positive association with HDL-C alongside an inverse association with VAT. This opposite-direction pattern highlights PON3 as a molecular node connecting two aspects of cardiometabolic health. Biologically, its positive coupling with HDL-C is consistent with PON3's structural and functional association with HDL particles. As an HDL-associated esterase/lactonase primarily synthesized in the liver, PON3 associates with HDL-C particles and protects lipids against oxidative modification, potentially preserving lipoprotein function. Concurrently, the inverse relationship with VAT is consistent with functional animal and molecular models showing that human PON3 overexpression attenuates diet-induced weight gain, fat accumulation, and systemic oxidative stress, whereas PON3 knockout increases susceptibility to visceral obesity.^15,16^ Together, these findings raise the possibility that circulating PON3 links lipoprotein function with visceral fat accumulation, although future functional and Mendelian randomization studies are needed to determine the causal direction of these relationships.

IL-32 was directly linked to HDL-C. This topological centrality is consistent with evidence identifying IL-32 as a prominent marker of adipose tissue inflammation and extracellular matrix remodeling in the context of obesity.^17^ Furthermore, because prior work has demonstrated that IL-32 levels vary with weight loss,^18^ its central position within our VAT-centered network highlights IL-32 as a potential molecular link between visceral adiposity, adipose tissue inflammation, and systemic lipid metabolism.

Agouti-related protein (AGRP) was directly linked to TG and occupied a central position within the network topology, consistent with its established role in energy balance.^19^ This connectivity is supported by the physiological role of AGRP in energy homeostasis, where it promotes hyperphagia, reduces energy expenditure, and has been associated with TG deposition in adipose depots.^20^ Notably, AGRP was also directly connected to multiple inflammatory proteins in our network. Together, these connections position AGRP at the intersection of visceral adiposity, lipid metabolism, and inflammatory signaling, suggesting that its network role may extend beyond hypothalamic appetite regulation to molecular pathways associated with metabolic dysfunction in the context of visceral adiposity.

Some limitations warrant consideration. First, the network integration approach applied here does not establish causality or directionality. Second, the predominantly older and White populations in VITAL and COSMOS may limit generalizability to younger and more diverse populations. Future studies incorporating more diverse populations and complementary omics data, as well as experimental approaches, will be important to further evaluate the biological mechanisms underlying the identified network architecture.

Overall, these findings identify visceral adiposity as a central organizing feature of the inflammatory proteomic landscape and provide a framework for understanding its molecular links with cardiometabolic traits. These findings also highlight visceral fat-related pathways as potential targets for future investigation.

## Figures and Tables

**Figure 1.**
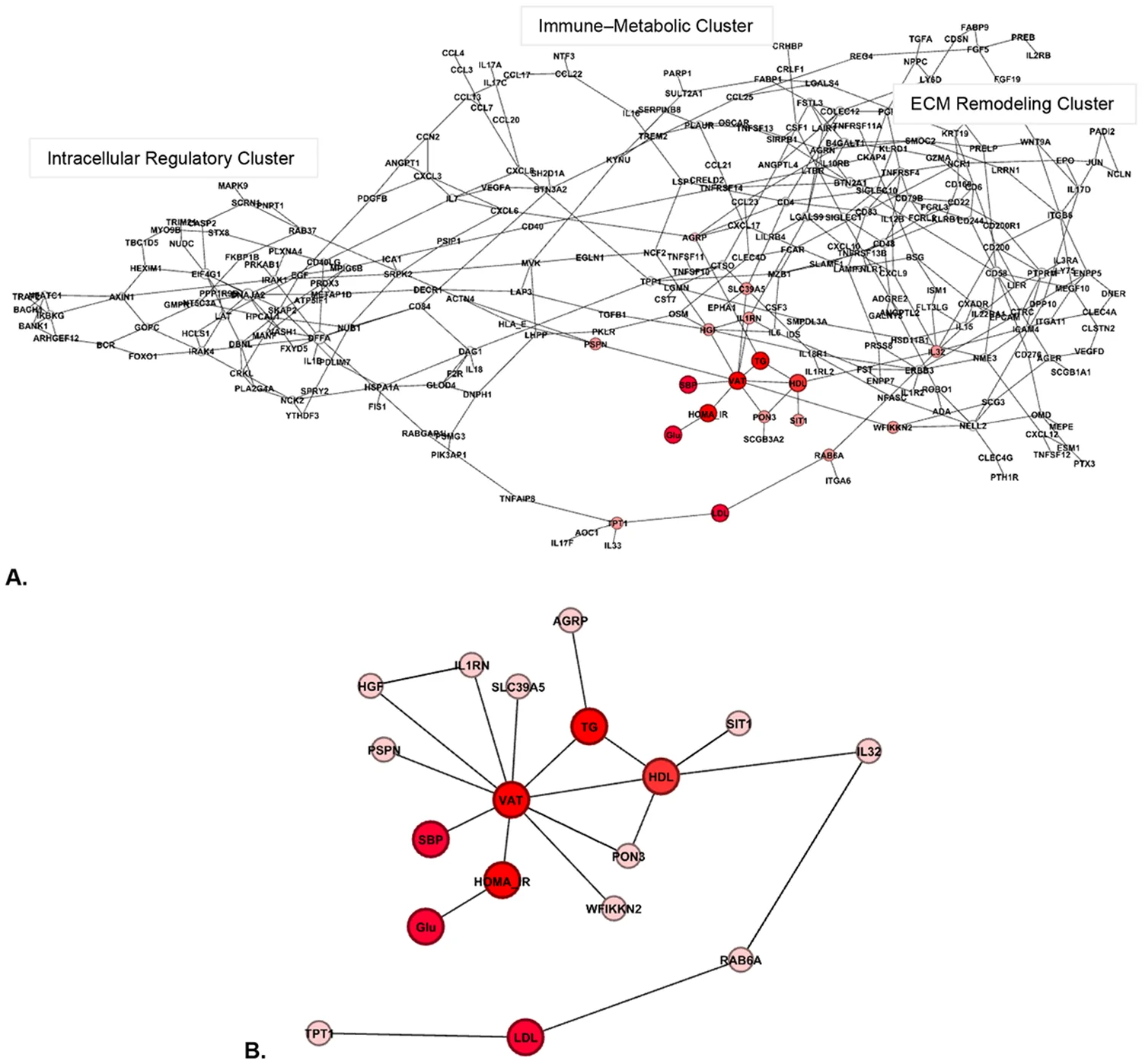
Network integration of inflammatory proteomics and cardiometabolic traits. Traits are depicted in red, and proteins directly connected to traits are depicted in pink. **A.** Inflammatory proteins are arranged into visually distinct subnetworks based on the ForceAtlas2 network layout. VAT emerged as a central network hub, exhibiting direct connections with multiple cardiometabolic traits and linking numerous inflammatory proteins to these traits. HDL-C displayed an additional VAT-independent connectivity pattern shared with LDL-C, involving proteins with functions related primarily to tissue architecture and extracellular matrix (ECM) remodeling. **B.** Expanded view of trait connectivity and proteins directly connected to each cardiometabolic trait.

**Figure 2.**
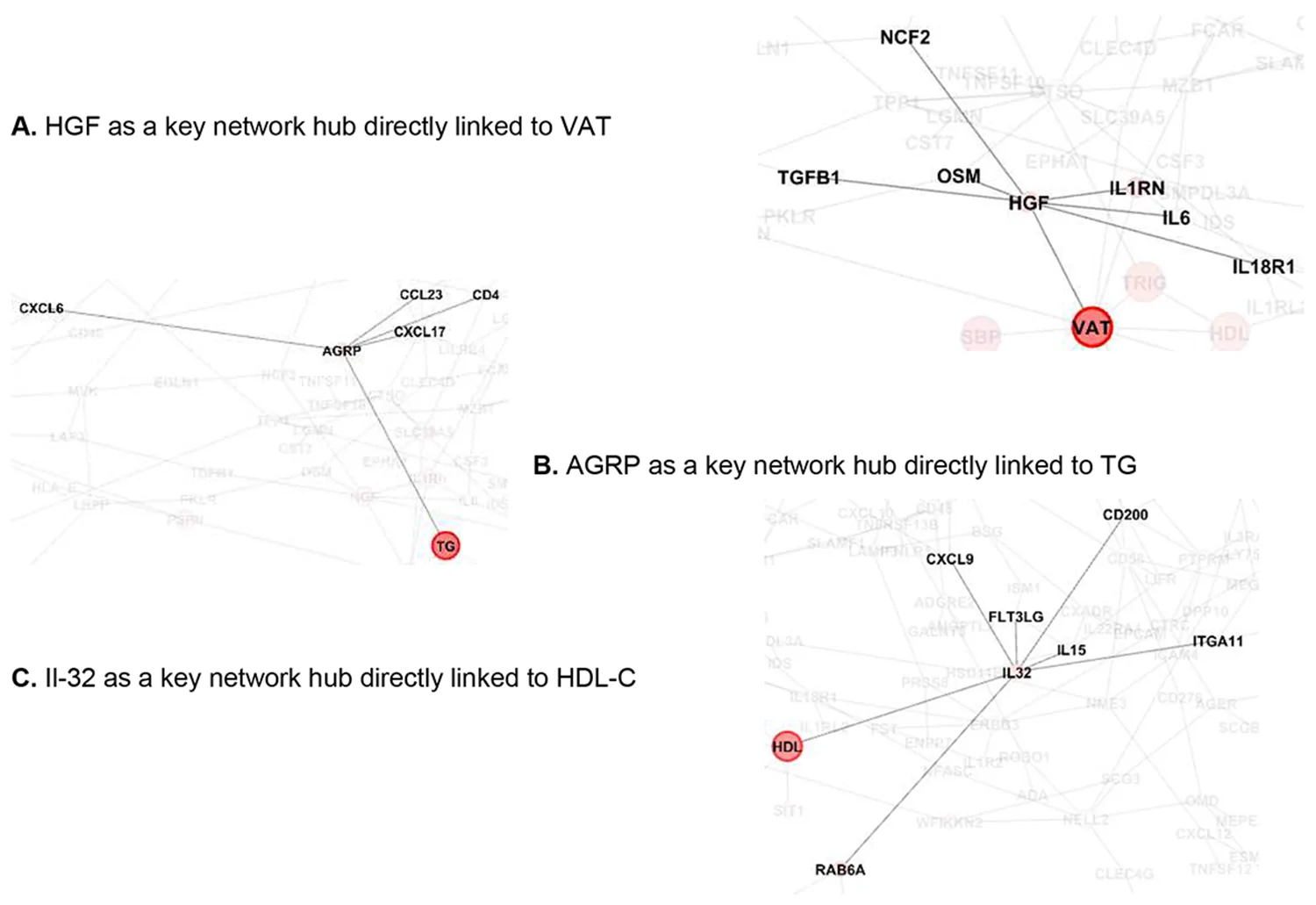
Expanded network neighborhoods of highly connected proteins directly associated with cardiometabolic traits. The displayed protein–protein connections represent direct network edges identified by conditional independence testing. **A.** HGF is directly associated with VAT. **B.** AGRP is directly associated with TG. **C.** IL32 is directly associated with HDL-C.

**Figure 3.**
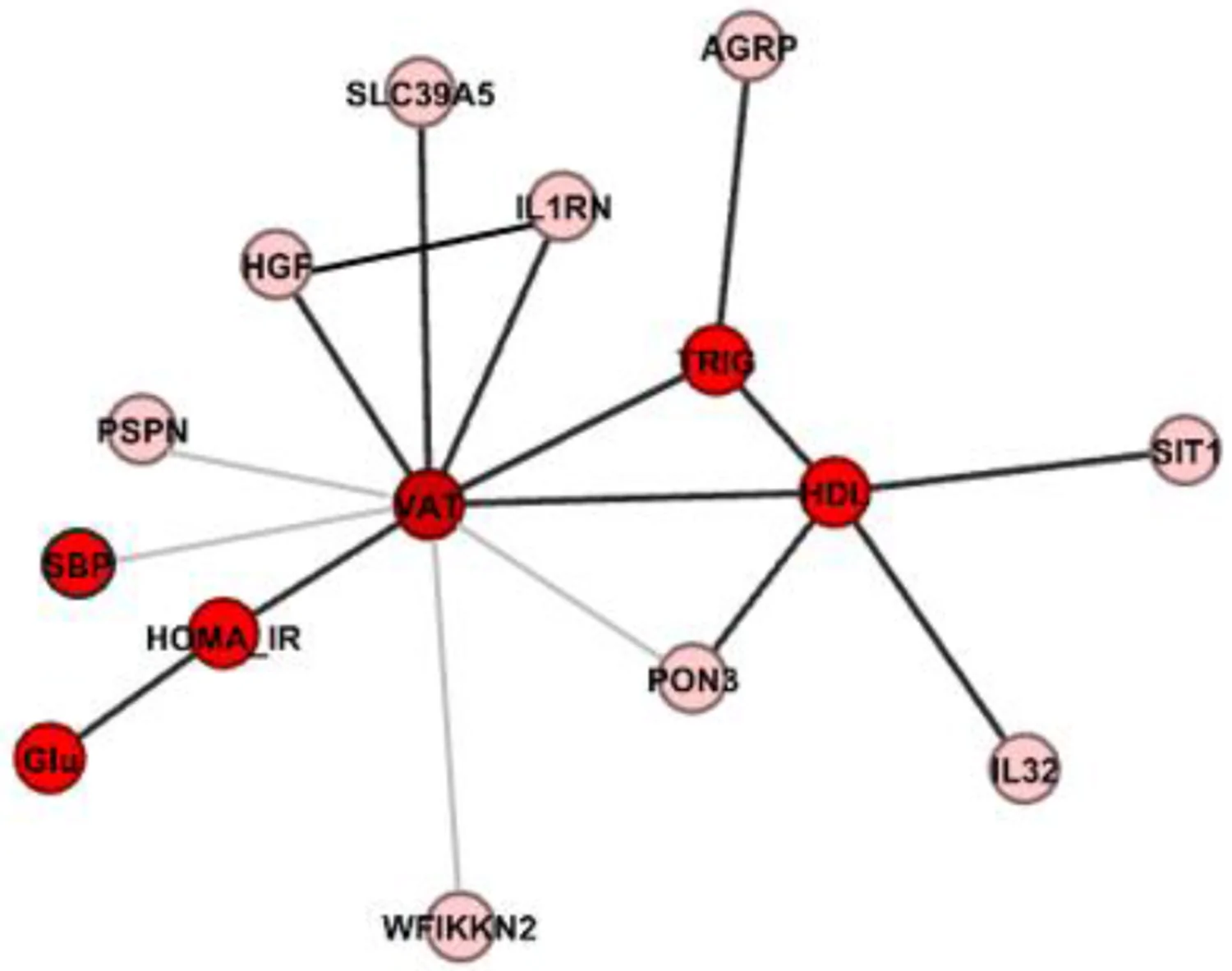
Replication of trait–trait and direct protein–trait network connections in the independent COSMOS cohort (n = 371). Dark edges indicate connections replicated at the significance threshold of *α* = 1 × 10^−3^, whereas gray edges denote connections identified in the discovery cohort that did not achieve statistical significance in the replication cohort.

**Figure 4.**
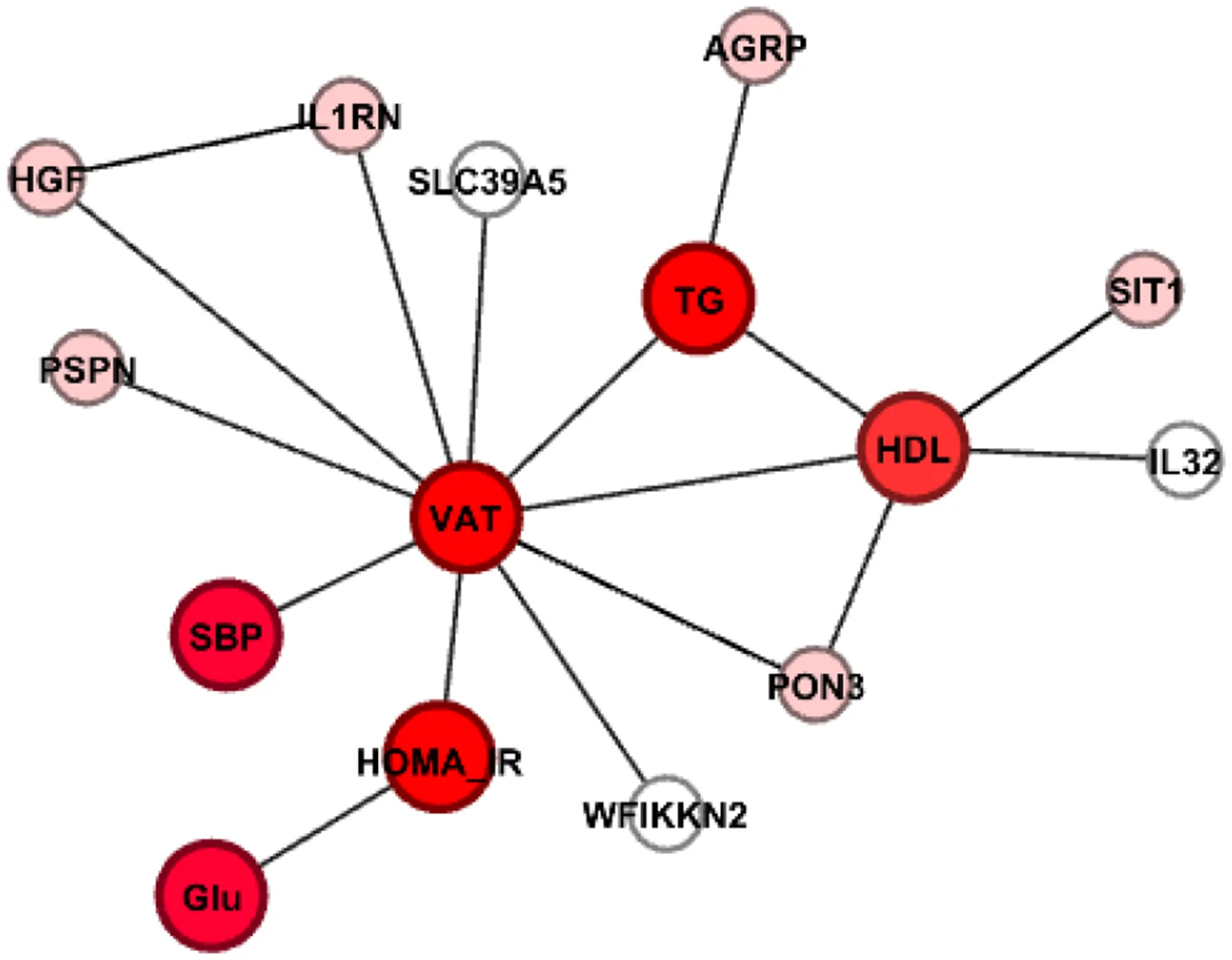
Longitudinal protein–trait network. Pink edges and nodes indicate significant within-person associations between changes in trait-linked protein levels and corresponding changes in clinical traits (P < 0.05). White nodes represent protein–trait connections for which longitudinal associations did not reach statistical significance.

**Figure 5.**
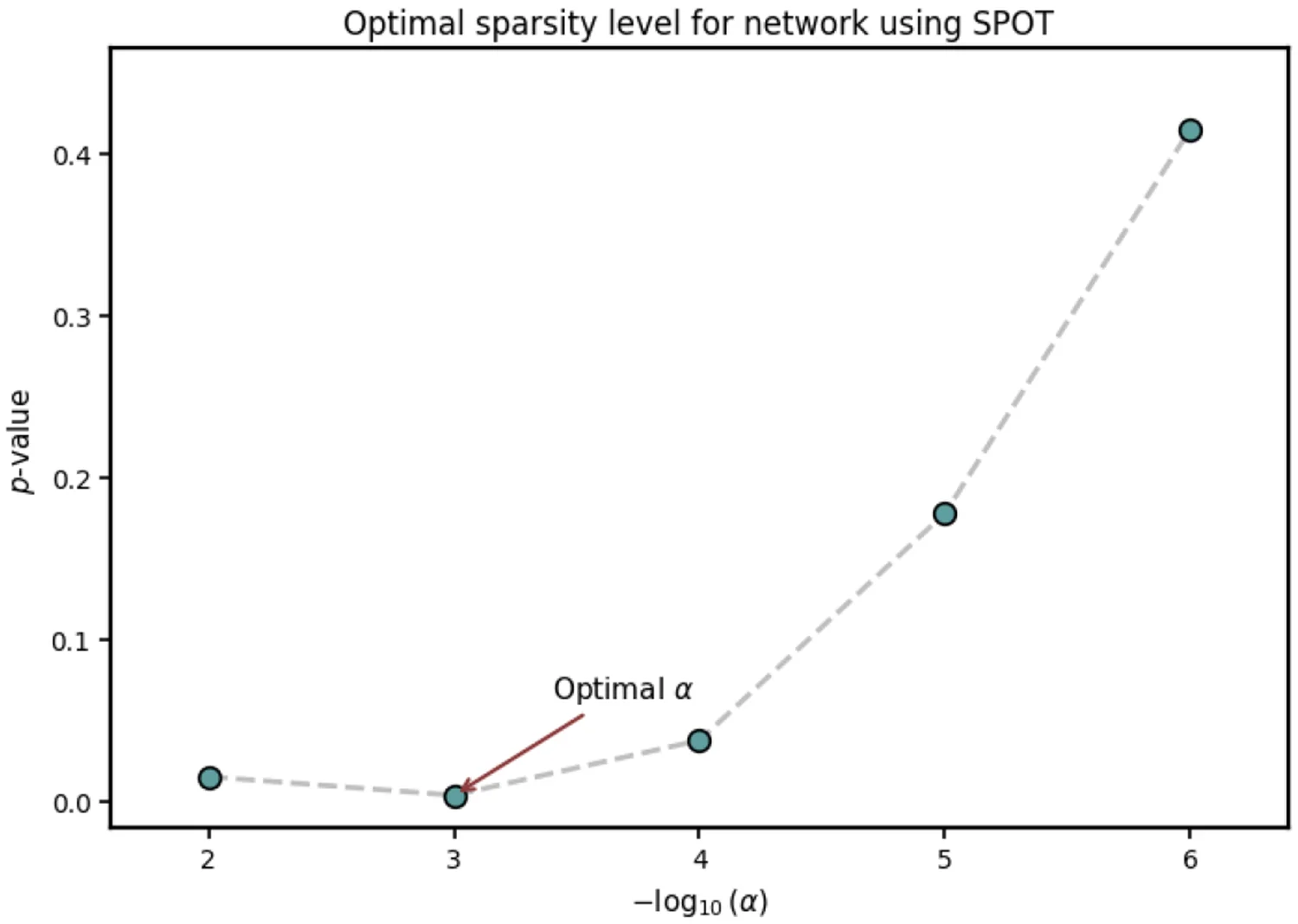
Optimization of the network sparsity parameter. Network topology metrics were evaluated across sparsity parameters ranging from α = 1 × 10^−1^ to 1 × 10^−7^. SPOT identified α = 1 × 10^−3^ as the optimal data-driven sparsity level, corresponding to the inflection point at which network topology changed most substantially, supporting the selected sparsity parameter.

**Table 1.** Baseline characteristics of participants in VITAL (the discovery cohort) and COSMOS (the replication cohort)

Characteristics	VITAL (n = 635)	COSMOS (n = 371)
Age (mean)	64±6.1	69±5.2
Female%	49	52
Body mass index (BMI, kg/m2)	28±4.8	28±5.3
Race%	86% White	96% White
Cardiometabolic traits, Median [Q1, Q3]		
Fasting glucose (mg/dL)	97 [92, 103]	98 [92, 104]
HOMA-IR (index)	1.8 [1.1, 2.7]	1.9 [1.1, 2.9]
HDL-C (mg/dL)	51 [42, 64]	60[49,74]
TG (mg/dL)	90 [67, 122]	86 [65,123]
SBP (mmHg)	122 [112,130]	130 [119, 140]
VAT (g) measured by DXA	741 [512,969]*	739 [483,1019]

* VAT in VITAL is measured only on 525 individuals out of 635 individuals.

**Table 2.** Effect sizes and statistical significance of proteins directly linked to clinical traits in the integrated networks. Connectivity indicates the number of direct protein–protein edges associated with each protein in the proteomic network and reflects its degree of network centrality.

Direct Connections	connectivity	effect size	SE	p-value
IL1RN - VAT	3	0.59	0.03	4 e-49
HGF - VAT	6	0.55	0.03	3 e-42
PON3 - VAT	1	− 0.49	0.03	8 e-34
WFIKKN2 - VAT	2	− 0.45	0.03	9 e-27
SLC39A5 - VAT	3	0.38	0.04	6 e-19
PSPN - VAT	2	0.39	0.04	1 e-14
PON3 - HDL	1	0.45	0.03	4 e-32
SIT1 - HDL	0	− 0.40	0.03	3 e-25
IL32 - HDL	5	0.32	0.03	1 e-14
AGRP - TG	4	0.64	0.03	2 e-61

**Table 3.** Within-person associations between changes in trait-linked proteins and corresponding trait changes.

Protein-Trait (Delta)	Effect Size	SE	p-value
IL1RN - VAT	0.42	0.10	4 e-5
HGF - VAT	0.67	0.17	1 e-4
PON3 - VAT	− 0.97	0.28	8 e-4
PSPN - VAT	0.21	0.08	2 e-2
WFIKKN2 - VAT	− 0.32	0.21	0.14
SLC39A5 - VAT	0.09	0.10	0.38
SIT1 – HDL-C	− 0.47	0.08	4 e-8
PON3 – HDL-C	1.44	0.28	4 e-7
IL32 – HDL-C	0.08	0.12	0.50
AGRP - TG	0.86	0.08	1 e-23
